# Association between physical activity patterns of working-age adults and social jetlag, depressive symptoms, and presenteeism

**DOI:** 10.1093/joccuh/uiae068

**Published:** 2024-11-13

**Authors:** Jaehoon Seol, Rina So, Fumiko Murai, Tomoaki Matsuo

**Affiliations:** Institute of Health and Sport Sciences, University of Tsukuba, 1-1-1 Tennodai, Tsukuba, Ibaraki 305-8577, Japan; International Institute for Integrative Sleep Medicine (WPI-IIIS), University of Tsukuba, 1-1-1 Tennodai, Tsukuba, Ibaraki 305-8577, Japan; Department of Frailty Research, Center for Gerontology and Social Science, National Center for Geriatrics and Gerontology, 7-430 Morioka-cho, Obu, Aichi 474-0038, Japan; Research Center for Overwork-Related Disorders, National Institute of Occupational Safety and Health, Japan (JNIOSH), 6-21-1 Nagao, Tama-ku, Kawasaki, Kanagawa 214-8585, Japan; Ergonomics Research Group, National Institute of Occupational Safety and Health, Japan (JNIOSH), 6-21-1 Nagao, Tama-ku, Kawasaki, Kanagawa 214-8585, Japan; Research Center for Overwork-Related Disorders, National Institute of Occupational Safety and Health, Japan (JNIOSH), 6-21-1 Nagao, Tama-ku, Kawasaki, Kanagawa 214-8585, Japan; Research Center for Overwork-Related Disorders, National Institute of Occupational Safety and Health, Japan (JNIOSH), 6-21-1 Nagao, Tama-ku, Kawasaki, Kanagawa 214-8585, Japan; Ergonomics Research Group, National Institute of Occupational Safety and Health, Japan (JNIOSH), 6-21-1 Nagao, Tama-ku, Kawasaki, Kanagawa 214-8585, Japan

**Keywords:** weekend warrior, exercise, circadian rhythm, mental health, work productivity, social jetlag

## Abstract

Objectives: This study aimed to evaluate the association of physical activity with social jetlag, depressive symptoms, and presenteeism.

Methods: This cross-sectional study included 8247 working-age adults (females, 44.6%; age, 20-64 years). Social jetlag was defined as the absolute difference between the midpoint of bedtime and wake time on workdays and free days. Depression symptoms were assessed using the Center for Epidemiologic Studies Depression Scale, and presenteeism was evaluated using the Work Functioning Impairment Scale. Exercise habits were classified into 4 groups based on the World Health Organization guidelines: nonactive (NA; *n* = 4223), insufficiently active (IA; *n* = 3009: exercise below guideline levels), weekend warriors (WW; *n* = 220: exercise 1-2 times per week meeting guideline levels), and regularly active (RA; *n* = 793: exercise at least 3 d/wk meeting guideline levels). Using multiple and Poisson regression analyses, we examined the association between exercise habits and each outcome.

Results: Social jetlag, depression, and presenteeism were more favorable with shorter sedentary times and longer durations of moderate- and vigorous-intensity exercise. Compared with the RA group, the NA group had a significantly higher prevalence of social jetlag (prevalence ratio [PR] = 1.30), depression (PR = 1.31), and presenteeism (PR = 1.35). The IA group had a significantly higher prevalence of depression (PR = 1.33) and presenteeism (PR = 1.38).

Conclusions: Exercising with a certain frequency and intensity may help prevent symptoms of depression and social jetlag, and consequently prevent presenteeism.

## Teaser

Key points: What is already known on this topic: Social jetlag is associated with depressive symptoms, thus lowering occupational performance. Although exercise can improve mental health, exercising only on nonworkdays may contribute to social jetlag.

What this study adds: Social jetlag, depression, and presenteeism were more favorable with shorter sedentary times and longer durations (3 or more days instead of 1 or 2) of moderate- and vigorous-intensity exercise.

How this study might affect research, practice, or policy: Encouraging exercise routines of at least 3 d/wk rather than 1 or 2 days (ie, weekends only) could contribute to productivity and mental health.

## 1. Introduction

Mistimed sleep, occurring during jetlag and shift work, desynchronized from the central circadian clock, adversely affects circadian transcripts in the human blood transcriptome; these adverse effects cause negative health outcomes, such as impaired mental health, cardiometabolic diseases, and mortality.^1^^-^^4^ One factor that contributes to mistimed sleep is social jetlag, which refers to the misalignment between an individual’s biological clock and their social obligations,^4^ typically seen in students and workers who rely on alarms to wake up and conform to societal norms.^4^ This disruption of the circadian rhythm further exacerbates adverse effects.^4^

An epidemiological study showed that working-age adults who have more than 2 hours of social jetlag have approximately twice the likelihood of experiencing depressive symptoms compared with those with less than 1 hour of social jetlag.^5^ Moreover, in addition to depressive symptoms, the adverse effects of social jetlag include lower occupational performance and productivity, such as presenteeism and/or absenteeism among the working generations.^6^ This is based on the disruption of nonphotic zeitgebers, which are responsible for regulating physiological circadian rhythms and can trigger episodes of depression (ie, the social rhythm hypothesis of depression).^7^ An experimental study showed that forced desynchrony (eg, a shift-work schedule or social jetlag) had more adverse effects than sleep restriction on the blood transcriptome of circadian rhythms.^1^

Exercise is an effective tool for improving mental health and circadian rhythms.^8^^,^^9^ Several systematic reviews have demonstrated the effects of exercise on mental health and circadian rhythms, and the mechanism has been partially proven.^8^^,^^9^ The effectiveness of exercise depends on a combination of intensity, frequency, and duration. Particularly for workers, exercise habits are influenced by how they spend their leisure time and engagement in less sedentary time.^10^ Humans have only 24 hours a day, and behaviors like sleep, sedentary time, and exercise are interdependent.^10^ Weekend warriors and regularly active individuals may reduce sedentary time, and replacing sedentary time with moderate-to-vigorous activity has been shown to improve mental health in workers.^10^ Recently, reports have investigated individuals who predominantly engage in exercise on weekends, referred to as “weekend warriors.” They meet the exercise criteria recommended by the World Health Organization (WHO) (at least 150 minutes of moderate-intensity or 75 minutes of vigorous-intensity exercise per week) for 1 or 2 days, and demonstrated comparable positive benefits on all-cause, cardiovascular disease, and cause-specific mortality.^11^^,^^12^ However, as noted in the comments on the previously reported study,^11^^,^^13^ there are arguments suggesting that engaging in exercise for only 1 or 2 days may contribute to social jetlag. The rationale behind this argument is that, if the absence of differentiation between free days and workdays is associated with reduced social jetlag, engaging in exercise exclusively on holidays could induce social jetlag.

Studies examining the effects of various exercise patterns on social jetlag, depressive symptoms, and presenteeism among workers are lacking. Furthermore, the association between social jetlag and depression and between depression and presenteeism has been reported,^5^^,^^14^ suggesting that the negative effects of social jetlag may affect presenteeism through depression in workers. As reported in these studies, social jetlag, depression, and presenteeism of workers are interrelated. However, few studies have specifically examined the relationship between distinct physical activity patterns, such as weekend warrior and regularly active lifestyles, and these important health factors for workers. Thus, this study aimed at investigating the relationship between physical activity patterns and social jetlag, depressive symptoms, and presenteeism among working-age adults.

## 2. Methods

### 2.1. Study design and participants

This cross-sectional study used data from a web-based survey of working-age adults in the Greater Tokyo Area of Japan. The survey was conducted between January and July 2018. Participants who agreed to participate in the study had to meet the following eligibility criteria: (1) aged 20-64 years, (2) employed in the Greater Tokyo area of Japan (Tokyo, Saitama, Chiba, and Kanagawa Prefectures), and (3) working at least 3 d/wk. We recruited a sample of 10 000 workers with diverse employment types based on the 2017 Japan Labour Force Survey composition ratio of employed individuals by sex, age, and industry type, as provided by the Ministry of Internal Affairs and Communications.^15^ We aimed to recruit exactly 10 000 participants, and the recruitment was conducted by the internet research company (IDEA PROGGET Co, Ltd, Tokyo) in a manner that allowed us to reach this target. If the initial recruitment fell short, additional participants were recruited to ensure the final sample size was exactly 10 000 workers, as planned. Although all participants completed the survey, a total of 1753 workers (17.5%) were excluded due to incomplete data, erroneous values, or outliers. Specifically, respondents who provided inconsistent or erroneous answers, such as reporting the same time for both wake-up and sleep times (*n* = 234), were excluded. Outliers were defined as data points that fell outside ±3 SDs from the mean (*n* = 767). Additionally, respondents who failed to select whether their wake-up or sleep times were in the am or pm, resulting in erroneous sleep duration calculations (*n* = 752), were also excluded. Ultimately, 8247 workers were included in the analyses.

This study was conducted according to the guidelines of the Declaration of Helsinki. The Ethics Committee of the National Institute of Occupational Safety and Health, Japan, reviewed and approved the study protocol (reference no. H2921). All participants provided web-based informed consent.

### 2.2. Measurements

Sleep behavior, including social jetlag and physical activity, was assessed using the Workers’ Living Activity-Time Questionnaire (WLAQ).^16^ The WLAQ consists of a worker’s 24-hour behaviors, including sleep, sedentary time, occupational-related activity, and leisure-time physical activity on workdays and free days. The WLAQ has demonstrated acceptable reliability and validity.^16^ The frequency, duration, and intensity of the coded physical activity data were used to determine if the minimum criteria were met with reference to previous studies.^11^ The WLAQ consists of 3 questionnaires with 2 patterns (workdays and free days) as follows: First, for “In your leisure time on workdays (or free days), how much intentional physical activity do you engage in?” participants choose from the following 4 categories: none/almost none (coded as “0 days”), 1-3 days per month (“0.5 days per week”), 1 or 2 days a week (“1.5 days per week”), and more than 3 days per week (“3 days per week”) on workdays. For free days, the options are none/almost none (“0 days per week”), 1-2 days per month (“0.5 days per week”), once a week (“1 day per week”), and more than 2 days per week (“2 days per week”). Second, for “Please provide the average exercise time per day,” participants choose from the following 4 categories: less than 15 minutes (“7.5 minutes”), 15-30 minutes (“22.5 minutes”), 31-60 minutes (“45 minutes”), and more than 60 minutes (“60 minutes”). Third, for “Please tell us the approximate intensity of the exercise per session,” participants choose from the following 3 categories: no sweating or panting (“low intensity”), sweating and panting (heart rate increase) (“moderate intensity”), or strained breathing to the point of exhaustion (“vigorous intensity”).

Social jetlag was calculated as the absolute difference between the midpoint of bedtime and wake time on workdays and free days.^17^ A large value indicates a significant disparity between workdays and free days. In this study, social jetlag ranged from 0 to 5.75 hours.

Depressive symptoms were assessed using the Japanese version of the Center for Epidemiologic Studies Depression (CES-D) scale.^18^ The CES-D measures depressive symptoms experienced in the past week. Responses were rated on a scale of 0-3 based on symptom frequency, with total scores in the range of 0-60.

Presenteeism was assessed using the Work-Functioning Impairment Scale (WFun).^19^ Its reliability and validity have been previously confirmed.^19^ The WFun evaluates responses to 7 questions, including statements such as: (1) “I haven’t been able to engage socially,” (2) “I haven’t been able to maintain the quality of my work,” (3) “I have had trouble thinking clearly,” (4) “I have taken more rests during my work,” (5) “I felt that my work isn’t going well,” (6) “I haven’t been able to make rational decisions,” and (7) “I haven’t been proactive about my work.” Each question is scored on a scale of 1-5, resulting in a total score ranging from 7 to 35 points, with higher scores indicating worse workability.

### 2.3. Potential confounders

To identify potential confounders, we referenced previous studies^5^^,^^11^ and included the following variables: age (continuous), sex (male or female), body mass index (BMI) (continuous), medication status (antihypertensive, lipid-lowering, antidiabetic, antihyperuricemic, antidepressants) (yes or no), occupational information (type of work, job type, employment status), annual income (less than 2 million Japanese yen, 2-4 million Japanese yen, 4-6 million Japanese yen, 6-8 million Japanese yen, 8-10 million Japanese yen, more than 10 million Japanese yen, or refused to answer), marital status (yes or no, or refused to answer), smoking history (past/never or current), drinking habits (never/less than 1-2 times per month, and more than 1-2 times per week), and average sleep duration on workdays and the free days (continuous). For BMI, participants reported their height and weight from their most recent health checkup conducted within the past year. Moreover, we calculated the estimated maximal oxygen consumption per unit time (e$\dot{\textrm{V}}$o_2max_), an indicator that allows the estimation of cardiorespiratory fitness, using the WLAQ.^16^^,^^20^

Occupational information data were originally classified into 12 categories based on the Japanese Standard Occupational Classification.^21^ Employment status (regular staff, part-time workers, contract employees [temporary workers, entrusted employees], employers) and work schedules (fixed-hour work, flex-time work, remote work) were also reported. Chronotype was calculated using the minimum sleep time on free days corrected for sleep debt on workdays (MSFsc).^22^ A higher MSFsc value represents a later chronotype, meaning the individual tends to wake up and go to bed later. Conversely, a lower MSFsc value indicates an earlier chronotype, with the individual waking up and going to bed earlier.^22^

### 2.4. Statistical analyses

Multiple regression analysis using the forced-entry method was employed to determine the association between average daily sedentary time and time spent in moderate and vigorous-intensity exercise for each dependent variable (social jetlag, depressive symptoms, and presenteeism). The adjusted variables included age, sex, BMI, marital status, smoking history, drinking habits, medication status, eV̇o_2max_, chronotype, average sleep duration on workdays and free days, exercise frequency, and occupational information, including employment status, work schedule, occupation type, and annual income. Participants were classified based on the following cutoffs: social jetlag (>2 hours),^5^ depressive symptoms (>16 points),^23^ and presenteeism (>14 points).^19^ Additionally, referring to a previous study, we defined 4 groups as follows: (1) the nonactive group (NA, *n* = 4223) were defined as not reporting any moderate- or vigorous-intensity physical activity; (2) the insufficiently active group (IA, *n* = 3009) were defined as reporting less than 150 min/wk of moderate-intensity physical activity and less than 75 min/wk of vigorous-intensity physical activity; (3) the weekend warriors group (WW, *n* = 220) were defined as reporting at least 150 min/wk of moderate-intensity physical activity or at least 75 min/wk of vigorous-intensity physical activity from 1 or 2 d/wk; and (4) the regularly active group (RA, *n* = 793) were defined as reporting at least 150 min/wk of moderate-intensity physical activity or at least 75 min/wk of vigorous-intensity physical activity from 3 or more sessions.^11^^,^^12^ To compare the characteristics of participants in the groups, we used a 1-way analysis of variance (ANOVA) with the Bonferroni post hoc test for continuous variables and the χ^2^ test for categorical variables. For categorical variables, dummy variables were created, and when significant differences were detected in the χ^2^ test, residual analysis was performed. For the main analysis, as the prevalence of each outcome exceeded 10%, we used Poisson regression analysis to investigate the relationship between physical activity patterns and the risk of social jetlag, depressive symptoms, and presenteeism, using the RA group as the reference. Additionally, to examine the effect of the weekend warrior on each dependent variable, Poisson regression analysis with the WW as the reference was conducted in the same manner. We calculated prevalence ratios (PRs) and 95% CIs. An unadjusted model, and 2 models were created to examine the effects of personal background factors and occupational information on each outcome. Model 1 was adjusted for age, sex, BMI, marital status, smoking history, drinking habits, medication status, estimated V̇o_2max_, chronotype, and average sleep duration on workdays and free days. Model 2 further incorporated adjustments for occupational information, including employment status, work schedule, occupation type, and annual income. By setting these models, we aimed to assess the impact of confounding factors in each model and to understand the influence of occupational information more thoroughly.

All analyses were performed using Python (version 3.12.2), which was built into Visual Studio Code (version 1.87.2). ANOVA, multiple regression analysis, and Poisson regression analysis used in this study were conducted using the *statsmodels* package, as well as the *scipy.stats* package for the χ^2^ test.

## 3. Results

**Figure 1:**
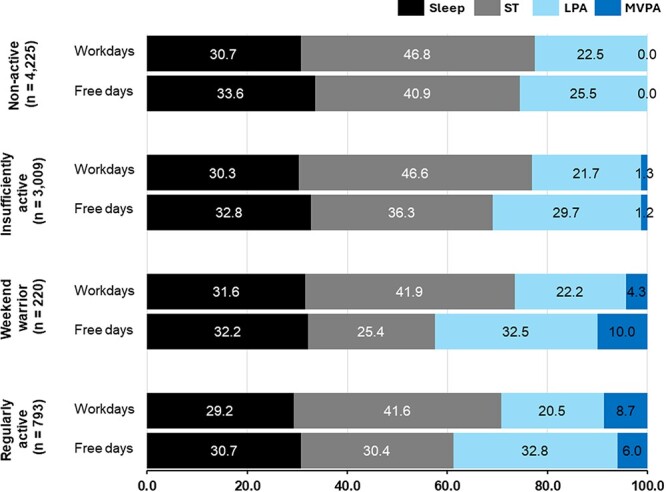
Percentage of sleep duration, sedentary behavior, and low-, moderate-, and vigorous-intensity physical activity on weekdays and weekends. Results obtained using the WLAQ, showing sleep duration, sedentary time, and physical activity by intensity on weekdays and weekends, expressed as percentages. LPA, low-intensity physical activity; MVPA, moderate- and vigorous-intensity physical activity; ST, sedentary time; WLAQ, Workers’ Living Activity-Time Questionnaire.

Table 1 presents the demographic characteristics of all the participants and physical activity patterns of the groups. The physical activity pattern exhibited certain characteristics in terms of age, chronotype, and eV̇o_2max_ (ANOVA; all *P* < .001), indicating significant differences among the 4 groups. In addition, significant differences were observed in sex, drinking habit, marital status, and antihypertensive use, as well as employment status and type of occupation (χ^2^ test; all *P* < .05), as shown in Table 1. Post hoc tests further revealed specific group differences, highlighting unique characteristics of each physical activity pattern. The prevalence was 1472 (17.8%) participants for social jetlag, 2797 (33.9%) for depression symptoms, and 2662 (32.3%) for presenteeism. The percentages of sleep, sedentary time, and physical activity by intensity showed small differences in sleep duration (+0.6% to 2.9%; 65-86 minutes) between weekdays and holidays, but there were higher fluctuations in sedentary time between workdays and free days, particularly marked by a decrease in the WW group (−16.5%; 92.3 minutes) and an increase in low-, moderate-, and vigorous-intensity exercise duration to compensate for this (Figure 1).

**Table 1:** Characteristics of the study participants.

**Variables**	**Inactive (1)** **(*n* = 4225)**	**Insufficiently active (2)** **(*n* = 3009)**	**Weekend warrior (3) (*n* = 220)**	**Regularly active (4)** **(*n* = 793)**	**ANOVA** ***P* value**	**χ** ^**2**^ ***P* value**	**Post hoc**
**Age, mean (SD), y**	43.4 (10.8)	43.3 (11.6)	43.4 (10.4)	45.2 (11.1)	<.001		4 > 1, 4 > 2, 4 > 3
**Female, *n* (%)**	2089 (49.4)	1179 (39.2)	119 (54.1)	290 (36.6)		<.001	1 > 2, 1 > 4, 3 > 2, 3 > 4
**BMI, mean (SD), kg/m** ^**2**^	22.3 (4.0)	22.4 (3.5)	21.8 (3.2)	22.4 (3.4)	.163		
**Sleep duration, mean (SD), h**	7.39 (1.49)	7.46 (1.46)	7.44 (1.51)	7.42 (1.56)	.290		
**Chronotype, mean (SD), h**	3.46 (1.52)	3.37 (1.48)	3.47 (1.48)	3.09 (1.56)	<.001		1 > 4, 2 > 4, 3 > 4
**Alcohol consumption (drinker), *n*(%)**	1853 (43.9)	1606 (53.4)	106 (48.2)	480 (60.5)		<.001	4 > 1, 4 > 2
**Current smoker, *n* (%)**	2022 (47.9)	1426 (47.4)	92 (41.8)	380 (47.9)		.372	
**Marriage status (yes), *n* (%)**	2301 (54.5)	1729 (57.5)	145 (65.9)	466 (58.8)		<.001	3 > 1, 3 > 2, 3 > 4
**eV̇o**_**2max**_ **, mean (SD), mL/kg/min**	37.7 (5.1)	39.8 (5.0)	40.7 (5.8)	41.2 (5.4)	<.001		4 > 1, 4 > 2, 4 > 3, 3 > 1, 3 > 2, 2 > 1
**Medication status**							
**Antihypertensive, *n* (%)**	351 (8.3)	307 (10.2)	13 (5.9)	63 (7.9)		.010	2 > 1, 2 > 3, 2 > 4
**Lipid-lowering, *n* (%)**	194 (4.6)	173 (5.7)	10 (4.5)	36 (4.5)		.354	
**Antidiabetic, *n* (%)**	152 (3.6)	111 (3.7)	3 (1.4)	29 (3.7)		.140	
**Antihyperuricemic, *n* (%)**	86 (2.0)	78 (2.6)	3 (1.4)	17 (2.1)		.344	
**Antidepressants, *n* (%)**	168 (4.0)	114 (3.8)	9 (4.1)	20 (2.5)		.265	
**Occupation categories**							
**Professional and technical, *n* (%)**	1029 (25.5)	799 (26.6)	47 (21.4)	213 (26.9)		.297	
**Administrative and managerial, *n*(%)**	72 (1.7)	93 (3.1)	5 (2.3)	26 (3.3)		<.001	2 > 1, 4 > 1
**Clerical, *n* (%)**	1368 (32.4)	1023 (34.0)	61 (27.7)	241 (30.4)		.075	
**Sales, *n* (%)**	415 (9.8)	449 (14.9)	32 (14.5)	122 (15.4)		<.001	4 > 1, 4 > 2, 4 > 3
**Service, *n* (%)**	659 (15.6)	320 (10.6)	46 (20.9)	83 (10.5)		<.001	1 > 2, 3 > 2, 3 > 4
**Security/protection, *n* (%)**	30 (0.7)	26 (0.9)	1 (0.5)	8 (1.0)		.715	
**Agricultural, forestry, fisheries, *n*(%)**	9 (0.2)	3 (0.1)	0 (0)	2 (0.3)		.566	
**Transports/communication, *n* (%)**	163 (3.9)	68 (2.3)	5 (2.3)	15 (1.9)		<.001	1 > 2, 1 > 4
**Production, construction, craft, *n* (%)**	208 (4.9)	98 (3.3)	4 (1.8)	36 (4.5)		.001	4 > 2
**Other, *n* (%)**	222 (5.3)	130 (4.3)	19 (8.6)	47 (5.9)		.013	3 > 2
**Leisure time physical activity**							
**No. of sessions per week (SD)**	0	1.7 (1.2)	2.0 (0.4)	4.1 (1.0)	<.001		3 > 1, 4 > 1, 3 > 2, 4 > 2
**Total MVPA time, mean (SD), min/wk**	0	37.1 (40.8)	208.7 (119.5)	218.3 (57.6)	<.001		2 > 1, 3 > 1, 4 > 1

Abbreviations: ANOVA, analysis of variance; BMI, body mass index; eV̇o_2max_, estimated maximal oxygen consumption per unit time^17^; MVPA, moderate-to-vigorous physical activity.

1 = Inactive group, 2 = Insufficiently active group, 3 = Weekend warrior group, 4 = Regularly active group. For categorical variables with significant χ^2^ results, pairwise comparisons were conducted using χ^2^ tests with Bonferroni correction. In the Post hoc column, notations such as “1 > 2” indicate that the Inactive group (1) has a significantly higher proportion than the Insufficiently active group (2) after correction. For continuous variables, ANOVA followed by Bonferroni correction was used for post hoc analysis, with each significant pairwise comparison explicitly listed in the Post hoc column.

**Figure 2:**
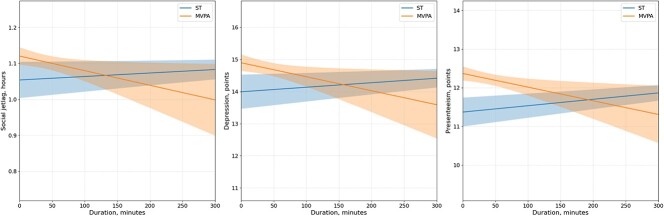
Relationship between social jetlag, depression, presenteeism, and sedentary behavior or exercise duration. This multiple regression analysis was adjusted for age, sex, BMI, occupational information, income, marital status, smoking history, drinking habits, medication status, estimated V̇o_2max_, chronotype, exercise frequency, and average sleep duration on workdays and free days. The figure illustrates regression lines and 95% CI for sedentary time and moderate- and vigorous-intensity exercise in relation to each dependent variable: social jetlag, depression, and presenteeism. Social jetlag, depression, and presenteeism worsen as their levels increase. BMI, body mass index; MVPA, moderate- and vigorous-intensity physical activity; ST, sedentary time; V̇o_2max_, maximal oxygen uptake per unit time.

After adjusting for potential variables and examining the association between sedentary time and moderate- and vigorous-intensity physical activity time for social jetlag, depression, and presenteeism, respectively, sedentary time was positively correlated (unstandardized coefficient, B = 0.001, 0.002, and 0.002, respectively; all *P* < .05) with social jetlag, depression, and presenteeism (Figure 2). In contrast, moderate- and vigorous-intensity physical activity time showed negative correlations (unstandardized coefficient, B = −0.001, −0.004, and −0.004, respectively; all *P* < .05) (Figure 2).

The prevalence of each condition by physical activity pattern was examined across the unadjusted model, Model 1 (which considered only demographic characteristics), and Model 2 (which added occupational information as an adjustment to Model 1) (Table 2). In all models, the PR showed a slight decreasing trend across the unadjusted models, Model 1, and Model 2, but the results were not significantly affected. Compared with the RA group, the NA group demonstrated a significantly higher prevalence of social jetlag (PR = 1.30; 95% CI, 1.06-1.60), depression (PR = 1.31; 95% CI, 1.13-1.53), and presenteeism (PR = 1.35; 95% CI, 1.15-1.58) in Model 2 (Table 2). The IA group showed a significantly higher prevalence of depression (PR = 1.33; 95% CI, 1.14-1.55) and presenteeism (PR = 1.38; 95% CI, 1.18-1.61) than the RA group in Model 2 (Table 2). The WW group showed no significant differences compared with the RA group in all models (Table 2).

**Table 2:** Association of physical activity patterns by participant characteristics and social jetlag, depressive symptoms, and presenteeism.

	**No. of prevalence, *n* (%)**	**Prevalence ratio (95% CI)** ^**a**^		
		**Unadjusted**	**Model 1**	**Model 2**
**Reference for the regularly active**				
***Social jetlag***				
**Regularly active**	108 (13.6)	1.00 (reference)	1.00 (reference)	1.00 (reference)
**Weekend warrior**	34 (15.5)	1.14 (0.77-1.67)	0.97 (0.66-1.43)	0.96 (0.65-1.41)
**Insufficiently active**	497 (16.5)	1.21 (0.99-1.49)	1.14 (0.93-1.41)	1.12 (0.91-1.39)
**Inactive**	830 (19.6)	**1.44 (1.18-1.76)**	**1.33 (1.08-1.63)**	**1.30 (1.06-1.60)**
				
***Depressive symptoms***				
**Regularly active**	197 (24.8)	1.00 (reference)	1.00 (reference)	1.00 (reference)
**Weekend warrior**	52 (23.6)	0.95 (0.70-1.29)	0.92 (0.68-1.25)	0.91 (0.67-1.24)
**Insufficiently active**	1055 (35.1)	**1.41 (1.21-1.64)**	**1.33 (1.14-1.55)**	**1.33 (1.14-1.55)**
**Inactive**	1487 (35.2)	**1.42 (1.22-1.64)**	**1.31 (1.13-1.53)**	**1.31 (1.13-1.53)**
				
***Presenteeism***				
**Regularly active**	184 (23.2)	1.00 (reference)	1.00 (reference)	1.00 (reference)
**Weekend warrior**	67 (30.5)	1.31 (0.99-1.74)	1.25 (0.94-1.65)	1.25 (0.95-1.66)
**Insufficiently active**	1022 (34.0)	**1.46 (1.25-1.71)**	**1.39 (1.19-1.63)**	**1.38 (1.18-1.61)**
**Inactive**	1389 (32.9)	**1.42 (1.22-1.65)**	**1.35 (1.16-1.59)**	**1.35 (1.15-1.58)**
				
**Reference for the weekend warrior**				
***Social jetlag***				
**Weekend warrior**	34 (15.5)	1.00 (reference)	1.00 (reference)	1.00 (reference)
**Inactive**	830 (19.6)	1.27 (0.90-1.79)	1.37 (0.97-1.93)	1.36 (0.96-1.92)
**Insufficiently active**	497 (16.5)	1.07 (0.76-1.51)	1.17 (0.83-1.66)	1.17 (0.93-1.67)
**Regularly active**	108 (13.6)	0.88 (0.60-1.30)	1.03 (0.70-1.51)	1.05 (0.71-1.54)
				
***Depressive symptoms***				
**Weekend warrior**	52 (23.6)	1.00 (reference)	1.00 (reference)	1.00 (reference)
**Inactive**	1487 (35.2)	**1.49 (1.13-1.96)**	**1.43 (1.09-1.89)**	**1.44 (1.09-1.90)**
**Insufficiently active**	1055 (35.1)	**1.48 (1.12-1.96)**	**1.45 (1.10-1.92)**	**1.46 (1.11-1.93)**
**Regularly active**	197 (24.8)	1.05 (0.77-1.43)	1.09 (0.80-1.48)	1.10 (0.81-1.49)
				
***Presenteeism***				
**Weekend warrior**	67 (30.5)	1.00 (reference)	1.00 (reference)	1.00 (reference)
**Inactive**	1389 (32.9)	1.08 (0.85-1.38)	1.08 (0.85-1.39)	1.08 (0.84-1.38)
**Insufficiently active**	1022 (34.0)	1.12 (0.87-1.43)	1.11 (0.87-1.42)	1.10 (0.86-1.41)
**Regularly active**	184 (23.2)	0.76 (0.58-1.01)	0.80 (0.61-1.06)	0.80 (0.60-1.06)

^a^Model 1: adjusted for age, sex, body mass index, marital status, smoking history, drinking habits, medication status, estimated maximal oxygen consumption per unit time, chronotype, and average sleep duration on weekdays and weekends. Model 2: Model 1 and further incorporated adjustments for occupational information, including employment status, work schedule, occupation type, and annual income. Bold indicates statistically significant differences compared with the reference.

When using the WW group as the reference, the NA (PR = 1.44; 95% CI, 1.09-1.90) and IA (PR = 1.46; 95% CI, 1.11-1.93) groups showed significantly higher prevalence in Model 2 of depression only (Table 2).

## 4. Discussion

This study examined the associations of various patterns of physical activity with social jetlag, depressive symptoms, and presenteeism among working-age adults. The results of this study support the findings and hypotheses of previous studies,^8^^,^^11^^-^^13^ which indicated better outcomes for social jetlag, depression, and presenteeism with reduced sedentary time and longer durations of moderate- and vigorous-intensity physical activity (Figure 2). Furthermore, regarding differences in physical activity patterns, the prevalence of depression and presenteeism was higher among those who did not exercise regularly compared with those who exercised regularly, especially among nonpractitioners, who had a higher prevalence of depression, presenteeism, and social jetlag (Table 2). In addition, we observed a lower prevalence of depression in the WW group compared with the IA and NA groups (Table 2).

Interestingly, whereas social jetlag, depressive symptoms, and presenteeism were negatively associated with sedentary time and positively associated with moderate-to-vigorous physical activity (Figure 2), the WW group, despite meeting the WHO recommendation of 150 minutes of activity per week, did not show better outcomes for social jetlag or presenteeism compared with the IA group, except for depressive symptoms. This could be due to the small sample size of the WW group, leading to low statistical power (eg, wide CIs for social jetlag in Table 2). However, the RA group, exercising almost daily, may be reducing presenteeism by relieving workplace stress.

Although the PR decreased slightly in the model adjusted for personal background factors (Model 1 in Table 2) compared with the unadjusted model, the results remained similar in the model that further adjusted for occupational information (Model 2 in Table 2). The influence of occupational information on the dependent variables (social jetlag, depressive symptoms, presenteeism) was nonsignificant.

As noted in previous studies,^13^ the WW physical activity pattern (exercising 1-2 d/wk) had no significant difference compared with the RA group in terms of social jetlag. A rat-model experiment revealed that a WW exercise schedule effectively reduced brain tissue myeloperoxidase activity and malondialdehyde levels. These reductions are associated with anti-inflammatory effects in depressive disorders.^24^ This reduction is similar to the effects of regular exercise.^24^^,^^25^ In fact, similar previous studies have already reported that WWs have a lower prevalence of depression.^24^^,^^25^ Although the style of exercise was not effective in reducing social jetlag and presenteeism in this study, our results may support the findings of previous studies that suggest the exercise habits of WWs are effective against depression.

The concept of the “weekend warrior” has gained attention due to its effectiveness, which is comparable to that of regular exercise habits, in preventing all-cause, cardiovascular, and cause-specific mortality as well as promoting mental health.^3^^,^^11^^,^^12^ Our results showed that the WW group had a significantly higher eV̇o_2max_ than the NA and IA groups (Table 1). eV̇o_2max_ is a reliable predictor of physical and mental health outcomes, including cardiovascular disease, mortality, and depressive symptoms, in individuals with or without chronic diseases.^26^^-^^29^ The eV̇o_2max_ levels in the WW group were similar to those in the RA group (Table 1). These results suggest that WW exercise is better than no exercise, but it may also be beneficial in reducing social jetlag and presenteeism.

Figure 1 suggests that differences in physical activity patterns between workdays and free days are more influenced by variations in sedentary activity and physical activity intensity than by sleep. The variances in physical activity patterns, which had been a concern in previous studies, did not have as large an impact on sleep habits but rather had a greater effect on sedentary activity and other activities. However, irregular sleep patterns can lead to reduced rapid eye movement sleep latency, increased overall rapid eye movement sleep duration, decreased slow-wave sleep, and heightened anxiety.^30^ These conditions may mediate depression and induce presenteeism in workers. Therefore, future research should examine the negative effects of differences in sleep habits, such as social jetlag and irregularity of bedtime and/or waking times, on depression and presenteeism.

Our study has a few limitations. First, the cross-sectional design limited the ability to establish causality. Therefore, future research should employ a longitudinal study design to examine causal relationships. Second, all variables were assessed using self-administered questionnaires, which may have led to underestimations or overestimations. For instance, the measurement of physical activity patterns using the WLAQ relied on self-reporting, which may have resulted in an underestimation of activity levels despite having a similar ratio of groups compared with a previous study.^11^ Furthermore, we did not collect data on the history of sleep disorders or the use of sleep medications. Therefore, we cannot rule out the possibility that sleep medications may have acted as a confounding factor in the results. To enhance the reproducibility of the findings, future studies should consider incorporating objective measurements such as those obtained using accelerometers. Finally, due to the smaller sample size compared with that of previous studies focusing on WWs,^11^^,^^12^^,^^25^ we were unable to classify participants by sex or occupation; hence, future studies should address this aspect.

## 5. Conclusion

Reducing sedentary time and increasing moderate- and vigorous-intensity physical activity in working-age adults may reduce the risk of social jetlag, depression, and presenteeism. Notably, compared with workers who exercised regularly, those who did not engage in exercise or were inactive had higher rates of depression and presenteeism. Additionally, individuals who did not exercise exhibited higher rates of social jetlag. However, engaging in exercise collectively on free days did not have a negative effect on social jetlag and may be more effective for preventing depression.

## Data Availability

The datasets used and/or analyzed during the current study are available from the corresponding author upon reasonable request (matsuo@h.jniosh.johas.go.jp).
